# Possible Novel Therapeutic Targets in Lymphangioleiomyomatosis Treatment

**DOI:** 10.3389/fmed.2020.554134

**Published:** 2020-09-24

**Authors:** Xixi Song, Hui Cai, Chengyu Yang, Xiaomin Xue, Jian Wang, Yuqing Mo, Mengchan Zhu, Guiping Zhu, Ling Ye, Meiling Jin

**Affiliations:** Department of Pulmonary and Critical Care Medicine, Zhongshan Hospital, Fudan University, Shanghai, China

**Keywords:** lymphangioleiomyomatosis, mTOR, pathogenesis, target, treatment

## Abstract

Lymphangioleiomyomatosis (LAM) is a rare systemic neoplastic disease that exclusively happens in women. Studies focusing on LAM and tuberous sclerosis complex (TSC) have made great progress in understanding the pathogenesis and searching for treatment. The inactive mutation of TSC1 or TSC2 is found in patients with LAM to activate the crucial mammalian target of rapamycin (mTOR) signaling pathway and result in enhanced cell proliferation and migration. However, it does not explain every step of tumorigenesis in LAM. Because cessation of rapamycin would break the stabilization of lung function or improved quality of life and lead to disease recurrent, continued studies on the pathogenesis of LAM are necessary to identify novel targets and new treatment. Researchers have found several aberrant regulations that affect the mTOR pathway such as its upstream or downstream molecules and compensatory pathways in LAM. Some therapeutic targets have been under study in clinical trials. New methods like genome-wide association studies have located a novel gene related to LAM. Herein, we review the current knowledge regarding pathogenesis and treatment of LAM and summarize novel targets of therapeutic potential recently.

## Introduction

Lymphangioleiomyomatosis (LAM) is a rare low-grade neoplasm that predominantly affects young and middle-aged women (1, 2). This disease appears as two forms. S-LAM occurs in 3.35–7.76/million women with an incidence of 0.23–0.31/million women/year (3). As a major feature of the clinical criteria to help diagnose tuberous sclerosis complex (TSC) (4), LAM occurs in 30–80% of women with TSC, which is the inherited form (TSC-LAM). TSC is another variable disease that affects the skin, brain, kidneys, lung, and heart, leading to multiple organ dysfunction. Cystic changes of pulmonary are also observed in 10–12% of males with TSC, but seldomly with symptomatic LAM. The pathogenic mutation in genes TSC1 or TSC2 is the definitive diagnostic criterion for TSC and related to the molecule etiology of LAM. LAM is characterized by progressively diffusive cystic destruction of lungs, which results in clinical symptoms including dyspnea, wheeze, cough, recurrent spontaneous pneumothorax, chylothorax, and a decline in expiratory flow rates (FEV_1_) and/or lung diffusion capacity (DLCO) (5). LAM lesions affect both pulmonary parenchyma and interstitium. The formation of the thin-wall cysts in parenchyma is associated with hyperplasia of type II pneumocytes and destructive changes in the elastic fibers and collagen (6). The structural changes may be related to “LAM cells.” The smooth muscle–like cells (LAM cells) in small clusters are observed on the edges of lung cysts and along blood vessels, lymphatics, and bronchioles in interstitium (7). These nodular LAM lesions are composed of more proliferative LAM cells in the center and less proliferative epithelioid cells at the periphery, and contain type II pneumocytes, lymphatic endothelial cells, and mast cells. The infiltration of LAM cells results in airway obstruction, vascular wall thickening, disruption of lymphatic vessels, venous occlusion, and hemorrhage with hemosiderosis (7). Extrapulmonary features consist of renal angiomyolipomas and lymphatic involvement such as lymphangioleiomyomas and chylous effusions (2, 8). Because of the rare nature and unremarkable symptoms as well as the chest radiograph, patients frequently tend to be misdiagnosed with asthma, COPD, idiopathic pulmonary fibrosis, or tuberculosis, and the delayed diagnosis may even come after decades (9). To make a clinical diagnosis of LAM, characteristic high-resolution CT (HRCT) feature—thin-wall cystic air sacs evenly distributed in bilateral lobes, surrounded by normal pulmonary tissues (9)—plus one or more of the following: presence of TSC, angiomyolipomas, chylous effusions, lymphangioleiomyomas, or elevated serum vascular endothelial growth factor-D (VEGF-D) ≥800 pg/ml, is required (10). When a definitive diagnosis is required in patients who have characteristic cysts on HRCT, but no additional confirmatory features (i.e., clinical, radiologic, or serologic), a transbronchial lung biopsy before a surgical lung biopsy is recommended (10). The diagnosis can be based on typical morphological appearance of LAM cells and positive staining for smooth muscle cell markers and human melanoma black-45 (HMB-45) by immunohistochemistry (10). The proliferation of LAM cells is commonly thought to be related to the mutational inactivation of gene TSC1 or TSC2 and subsequently abnormal activation of mTOR signaling pathway—the target of rapamycin and its analogs which become the only drugs for LAM treatment currently.

## The Central Role of the mTOR Signaling Pathway in Pathogenesis of LAM

**Figure 1:**
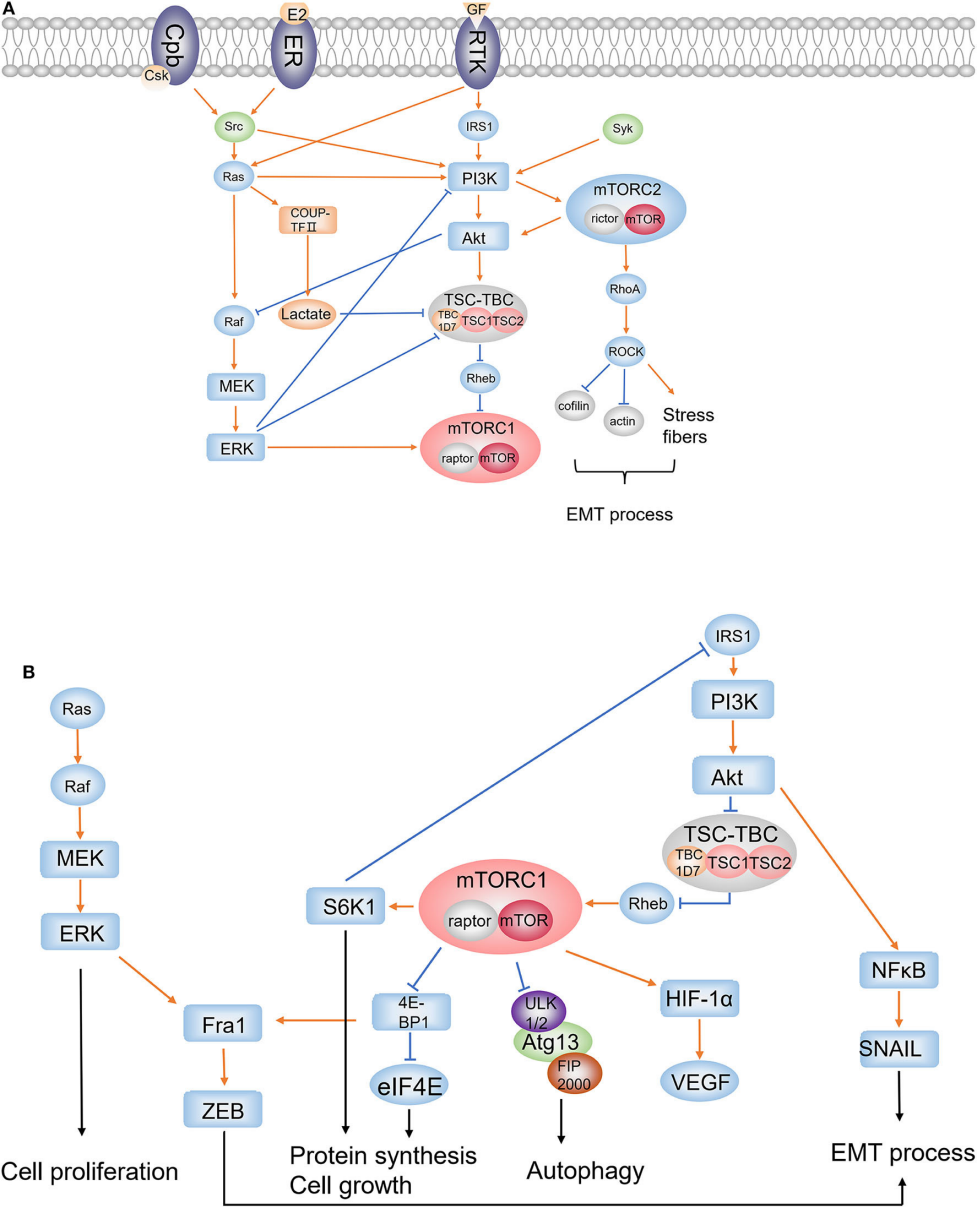
PI3K/Akt/mTORC1 pathway, Ras/Raf/MEK/ERK pathway, mTORC2 signaling, and the EMT process. **(A)** Upstream regulators and accompanying pathways affecting mTORC1; **(B)** downstream effectors regulated by mTORC1. (1) mTORC1 and mTORC2: rictor and mTOR are components of mTORC2, whereas raptor is the binding protein of mTORC1. (2) RTKs: extracellular growth factors bind to RTK and activate IRS1 and Ras. (3) Src: Src is activated by ER or Csk; Src activates PI3K to regulate PI3K/Akt cascade and activates Ras to affect ERK pathway and PI3K/Akt cascade. (4) PI3K/Akt/mTORC1: the PI3K/Akt cascade is activated by IRS1, Src, Ras, and Syk; Akt suppresses TSC1/2 complex, which downregulates mTORC1 through Rheb, to upregulate mTORC1; mTORC1 phosphorylates S6K1 and 4E-BP1 to promote protein synthesis and cell growth, and phosphorylates ULK1 to inhibit autophagy; S6K1 inhibits IRS1 to form a feedback loop in PI3K/Akt/mTORC1 pathway. Activation of mTORC1 results in accumulation of HIF-1α which increases the expression of VEGF. (5) Ras/Raf/MEK/ERK: Ras activates Raf; as a result, activated MEK1/2 upregulates ERK to affect cell proliferation. It is an important compensatory pathway of the PI3K/Akt/mTORC1 pathway. (6) The cross-regulation of Ras/Raf/MEK/ERK and PI3K/Akt/mTORC1: ERK inhibits PI3K and promotes mTORC1 directly or through repressing TSC1/2 complex. PI3K/Akt/mTORC1 negatively regulates Ras/Raf/MEK/ERK pathway by Akt inhibiting Raf. (7) mTORC2: PI3K activates mTORC2, which in turn activates Akt; RhoA is activated by mTORC2 and activates ROCK to induce the inhibition of cofilin and actin, and the increase of stress fibers; RhoA signaling plays an important role in cell migration and invasiveness through EMT process. (8) EMT: ERK pathway and the constitutive mTORC1 pathway converge on the Fra1–ZEB-1/2 transcriptional network to promote migration and invasion; NFκB activated by Akt induces SNAIL1 stabilization which plays an integral role throughout EMT. (9) NR2F2: activated RAS upregulates COUP-TFII and increases lactate production; overproduction of lactate disrupts the interaction of TSC2 and Rheb to increase mTORC1 activity.

Loss of heterozygosity for TSC genes, mostly TSC2, is found in somatic tissues either from women with S-LAM or patients with TSC-LAM who also carry germline TSC1 or TSC2 gene mutations (11). The TSC1 encodes hamartin, whereas the TSC2 encodes tuberin (12). Two proteins compose a tumor suppressor complex TSC1/2 which interacts with dozens of proteins. Tre2-Bub2-Cdc16 (TBC) 1 domain family, member 7 (TBC1D7) is one of the interacting proteins, which stably binds to the TSC1/2 heterodimer to form the TSC1-TSC2-TBC1D7 (TSC–TBC) complex (13). The TSC–TBC complex receives growth signals and suppresses the activity of small GTPase Ras homolog enriched in brain (Rheb) through the GAP domain of tuberin (1, 14), resulting in an inhibition effect on mTOR, a highly conserved serine–threonine kinase that plays an important role in the regulation of cell growth and proliferation (Figure 1). Generally, activated mTOR phosphorylates ribosomal S6 kinase 1(S6K1), Ras homolog gene family, member A (RhoA), and eukaryotic initiation factor 4E binding protein 1 (4E-BP1), leading to the activation of S6K1 and RhoA, and the inhibition of interaction between 4E-BP1 and eukaryotic initiation factor 4E (elF4E). Then, these downstream molecules result in enhancement of translation, cell growth, and proliferation, as well as cell survival and migration (15) (Figure 1). In LAM, inactivation mutation of TSC2 leads to absence of tuberin and loss of suppression to mTOR, ultimately facilitating the cell growth and proliferation.

Upstream signaling, including AKT, ERK1/2, RSK1, MK2, AMPK, GSK3, IKKβ, CDK1, and PLK1, regulates the mTOR pathway mainly through directly phosphorylating complex TSC1/2 (16). Particularly, the phosphoinositide 3-kinases (PI3K)–dependent signaling serves to inhibit the tuberin–hamartin heterodimer through direct protein kinase B (Akt)–dependent phosphorylation of tuberin at Ser-939 and Thr-1462 (the major Akt phosphorylation sites) (17) (Figure 1). The PI3K/Akt cascade was activated by insulin receptor substrate 1 (IRS1) when extracellular growth factors bind to receptor tyrosine kinase (RTK) (Figure 1). Coincidentally, the downstream target of mTOR, S6K1, repressed IRS1, thus forming a negative feedback loop in PI3K/Akt/mTOR axis, which is a regulatory strategy common to many signaling pathways (18) (Figure 1). Moreover, there exists another mechanism in which the PI3K signaling regulates the Akt/mTORC1 axis through activating mTORC2, which in turn modulates the phosphorylation and stability of Akt (19, 20) (Figure 1).

## Rapamycin Is an Inhibitor of mTORC1 But Not a Perfect Cure for LAM

As the catalytic subunit, mTOR exists in two complexes called mTOR complex 1 (mTORC1), the rapamycin-sensitive complex which controls cell-autonomous growth and responds to diverse extracellular signals including growth factors and nutrients, and mTORC2, which is insensitive to rapamycin and regulates key aspects of cell proliferation, including assembly of the actin cytoskeleton and cellular survival (14, 21). Rapamycin binds to the cytosolic protein FK506-binding protein 12 (FKBP12) which directly binds to mTORC1, resulting in the dissociation of raptor, which is another important component in complex 1, and inhibition of mTORC1 (5). Apart from lung transplantation, sirolimus (a rapamycin analog) is now U.S. FDA approved for the treatment of LAM based on several clinical studies and especially a landmark randomized controlled trial. The study demonstrates that sirolimus stabilizes lung function decline and improves quality of life and functional performance in patients with LAM (22–24). Although the efficacy and safety of sirolimus and everolimus are still being studied (Table 1), now, rapamycin is recommended as the first-line treatment option for qualified patients in the LAM guideline document (10).

**Table 1:** Current clinical trials studying potential targets for LAM.

**Target**	**Targeting therapy**	**Clinical trial**	**ClinicalTrials.gov identifier**	**Phase**
mTORC1	Sirolimus	Multicenter interventional lymphangioleiomyomatosis (LAM) early disease trial	NCT03150914	III
	Sirolimus and everolimus	Multicenter international durability and safety of sirolimus in LAM trial	NCT02432560	
Autophagy	Resveratrol	Resveratrol and sirolimus in lymphangioleiomyomatosis trial	NCT03253913	II
RTKs	Nintedanib	A pilot study of nintedanib for lymphangioleiomyomatosis (LAM)	NCT03062943	
Src	Saracatinib	Safety and efficacy of saracatinib in subjects with lymphangioleiomyomatosis	NCT02737202	II
RhoA	Simvastatin	The safety of simvastatin (SOS) in patients with pulmonary lymphangioleiomyomatosis (LAM) and with tuberous sclerosis complex (TSC)	NCT02061397	II

However, rapamycin is far from a perfect cure for LAM treatment according to serial discoveries. Most importantly, the monotherapy of sirolimus does not eliminate tumors despite the size reduction of solid proliferative lesions. On cessation of rapamycin therapy, the renal tumors regrow and lung function level even decreases to around baseline. Thus, as the only drug for LAM at the moment, rapamycin requires lifelong use, but it comes with a risk of remarkable adverse effects and possible acquired rapalog resistance (16). Moreover, LAM patients with lymphatic involvement may respond to sirolimus with a greater improvement in lung function than those without lymphatic disease, indicating that not all patients respond to rapamycin therapy in the same way (25, 26). These limitations of rapamycin monotherapy may be explained by its nature which is mainly cytostatic rather than pro-apoptotic and its highly selective specificity to mTORC1, leaving rapamycin-insensitive molecules like mTORC2 continuing to function. In addition, rapamycin promotes autophagy by re-activating RTKs and Akt through a negative feedback loop; not to mention the function of compensatory pathways who also promote cell growth (27, 28). Therefore, novel therapies for LAM and more specific studies about tumorigenesis are in an urge.

## Autophagy

### Rapamycin Promoting Autophagy and Cell Survival

The status of mTORC1 as a key inhibitor of autophagy may help explain why rapamycin requires lifelong usage and its limited efficacy. Autophagy is a catabolic process in which damaged proteins and organelles are degraded in lysosomes and recycled to provide metabolic precursors (29). This feature has given autophagy a critical effect in tumorigenesis. Three vital genes, unc51-like autophagy activating kinase (ULK), autophagy-related protein 13 (Atg13), and focal adhesion kinase family interacting protein of 200 kDa (FIP200), were required to initiate autophagy through the product ULK1/2-Atg13-FIP200 complex which is suppressed when mTORC1 phosphorylates ULK1 directly (5) (Figure 1). Through this pathway, rapamycin-induced inhibition of mTORC1 might increase the kinase activity of ULK1 to enhance autophagy. In an *in vitro* study, the level of hypoxia-induced autophagy was found lower in Tsc2^−/−^p53^−/−^ MEFs than in Tsc2^+/+^p53^−/−^ MEF; when treated with rapamycin, Tsc2^−/−^ p53^−/−^ MEFs showed increased autophagy marker LC3-II and decreased ubiquitin-binding protein p62/Sequestosome-1 (SQSTM1), the autophagy substrate (30). Another study observed fewer renal tumors in Tsc2^+/−^ Beclin 1^+/−^ mice than in Tsc2^+/−^ mice and extensive central necrosis of xenograft tumors, indicating that downregulated autophagy level inhibited cell survival in TSC-related tumor (31). One hypothesis is that autophagy is a protective mechanism for survival because it promotes the removal of damaged mitochondria thereby lowering levels of reactive oxygen species (ROS) in a metabolic stressed environment including nutrient deprivation, hormone stimulation, and hypoxia (29, 32). As a result of hyperactive mTORC1, low levels of autophagy in TSC2-null LAM cells limit their survival in the circumstance of bioenergetic stress. In tumor tissues, nutrients, and oxygen tend to be more insufficient in the inner region, which is exactly a natural bioenergetics stress (1). Thus, although it seems contrary, mTOR inhibitors restrain LAM cell growth while very likely benefit cell survival by promoting autophagy. It is obvious that extensive autophagy leads to cell death, so more questions about rapamycin and autophagy need to be answered, but the therapeutic potential of autophagy inhibitors has already shown its attractiveness in LAM treatments.

### Therapeutic Potential of Autophagy Inhibitors in the Treatment of LAM

Chloroquine and hydroxychloroquine are known as autophagy inhibitors, mainly used to treat malaria. Their effects in LAM have been revealed in *in vivo* and *in vitro* studies in which chloroquine inhibited TSC2-deficient cell survival and reduced xenograft tumor size to 60% (30). The effect was even more significant when chloroquine is combined with rapamycin than monotherapy of either. Based on these results, a clinical trial in patients with LAM is conducted to examine the safety, adverse effects, and efficacy of combined use of sirolimus and hydroxychloroquine (33) (Table 2). Results of phase I revealed that hydroxychloroquine in combination with sirolimus increased post-bronchodilator FEV_1_ (ml) significantly and decreased VEGF-D levels significantly during therapy. The walk distance in the 6-min walk distance test also increased significantly at the end of the treatment phase compared with the screening visit, and no serious adverse effect related to study drugs was reported. Nevertheless, patients with angiomyolipoma did not report any significant change in tumor size from baseline, the same with DLCO levels and St. George's Respiratory Questionnaire scores in all patients. Even the benefits went back to around baseline levels in the observation phase, like the consequence in sirolimus monotherapy. Considering the fact that only 14 patients were enrolled, and several have withdrawn, larger phase II/III trials are needed to further establish the long-term effectiveness of the combination therapy (33).

**Table 2:** Completed clinical trials studying therapeutic targets for LAM.

**Target**	**Targeting therapy**	**Clinical trial**	**ClinicalTrials.gov identifier**	**Completion date**
mTORC1	Sirolimus	Rapamycin therapy for patients with tuberous sclerosis complex and sporadic LAM	NCT00457808	March 2006
	Everolimus	A study to determine the effectiveness of escalating doses of RAD001 (everolimus) in patients with lymphangioleiomyomatosis	NCT01059318	June 2012
		RAD001 therapy of angiomyolipomata in patients with TS complex and sporadic LAM	NCT00457964	July 2013
		Long term follow up for RAD001 therapy of angiomyolipomata in patients with tuberous sclerosis (TSC) and sporadic lymphangioleiomyomatosis (LAM)	NCT00792766	September 2013
		Efficacy and safety of RAD001 in patients aged 18 and over with angiomyolipoma associated with either tuberous sclerosis complex (TSC) or sporadic lymphangioleiomyomatosis (LAM)	NCT00790400	November 2015
mTORC1 and autophagy	Sirolimus and hydroxychloroquine	Safety study of sirolimus and hydroxychloroquine in women with lymphangioleiomyomatosis (SAIL)	NCT01687179	August 2015
	Octreotide	Treatment with octreotide in patients with lymphangioleiomyomatosis	NCT00005906	April 2008
MMP	Doxycycline	Doxycycline in lymphangioleiomyomatosis (LAM)	NCT00989742	January 2013
Aromatase	Letrozole	Trial of aromatase inhibition in lymphangioleiomyomatosis (TRAIL)	NCT01353209	September 2014
Src	Saracatinib	The tolerability of saracatinib in subjects with lymphangioleiomyomatosis (LAM) (SLAM-1)	NCT02116712	July 2015
PDGFR	Imatinib mesylate	LAM pilot study with imatinib mesylate	NCT03131999	March 2019

Another medicine that inhibits autophagy and raises much attention is resveratrol, which may suppress the Akt negative feedback loop to function. It was demonstrated in *in vivo* studies that combination therapy of rapamycin with resveratrol blocked autophagy and induced apoptosis in TSC2-null cells (34). In *in vivo* and *in vitro* studies, the combination therapy prevented rapamycin-induced upregulation of Akt while maintaining inhibition of S6K1 signaling, which means it keeps suppressing the hyperactivation of mTORC1 (35). Moreover, resveratrol is well-tolerated with a low toxicity profile, so it may be worthy of further study. A clinical trial has been set up to study the potential benefit of resveratrol in combination with sirolimus (Table 1).

### Another Target in Autophagy

**Figure 2:**
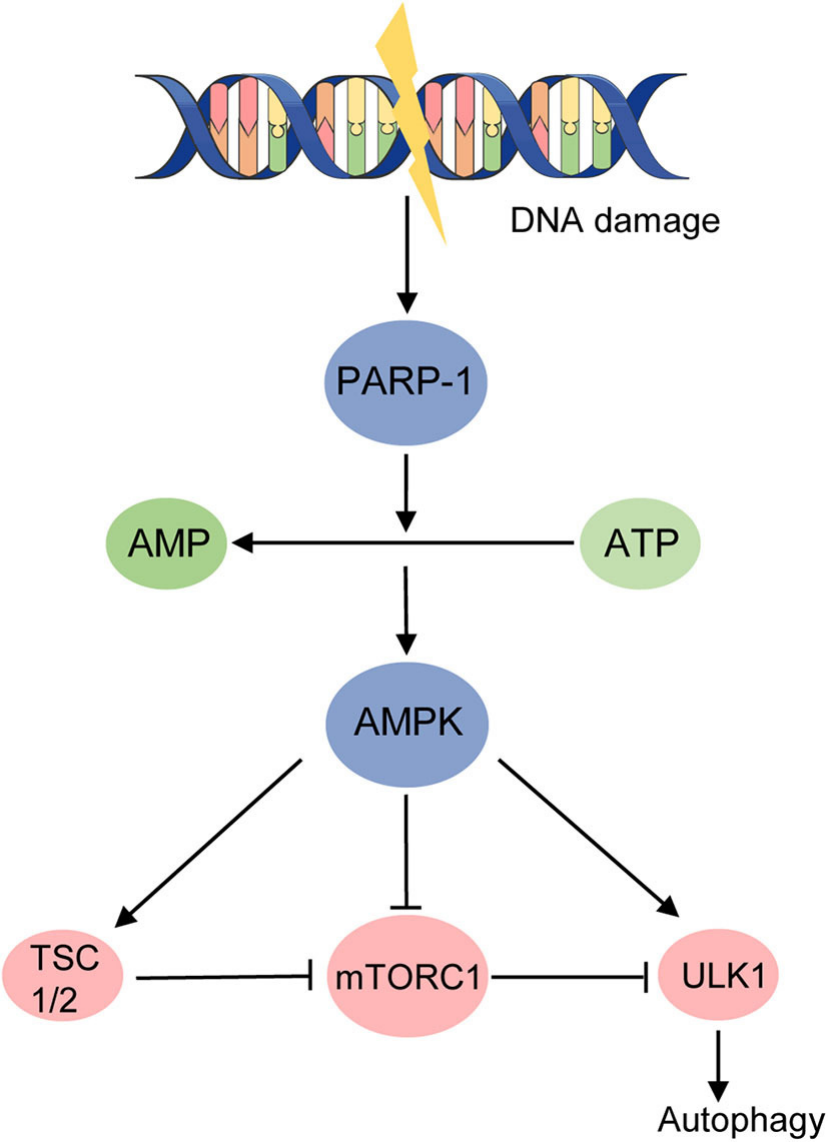
DNA damage activates autophagy in an energy starvation environment. AMPK activates autophagy in three mechanisms during energy starvation: (1) binding to and activating ULK1 through direct phosphorylation; (2) inhibiting mTORC1 directly; (3) activating TSC2 to suppress mTORC1. Hyperactivated PARP-1 induced by DNA damage raises the ratio of AMP to ATP and activates AMPK, ultimately promoting autophagy.

When studying the regulation of ULK1 and mTORC1 autophagy pathway, 50-AMP-activated protein kinase (AMPK) is found to be an important player. During energy starvation, AMPK could activate autophagy in three mechanisms: binding to and activating ULK1 through direct phosphorylation, inhibiting mTORC1 directly, or through activating TSC2 to suppress mTORC1 (36) (Figure 2). A nuclear protein, Poly (ADP-ribose) (PAR) polymerase (PARP)-1, was found to change the ratio of AMP to ATP which represents energy depletion and could be sensed by AMPK (Figure 2). Hyperactivated PARP-1 in response to ROS-induced DNA damage causes a depletion of ATP and activation of AMPK, inhibiting mTOR via TSC1/2 complex, ultimately inducing autophagy (29) (Figure 2). Upregulated PARP-1 expression was also found in TSC2-null cells derived from patients with LAM, as well as in TSC2-null xenograft tumors, renal tumors from TSC2 heterozygous mice, human angiomyolipomas, and LAM nodules (37). Studies then found the PARP-1 inhibitors suppress the growth and survival of TSC-null cells from LAM patients but not in a synergistic way with rapamycin *in vivo* (37). This study indicates the potential of targeting PARP1 in LAM therapy through PARP-1 inhibitors. However, although the PAR has been identified for over 50 years, serving as an initial sensor and mediating the early recruitment of DNA brake repair, it is not until recently that the anti-cancer effect of PARP-1 inhibitors was under emerging study, mainly in treating breast cancer (BRCA)–deficient tumors (38). Nevertheless, a recent study reported a contrary effect that the cytotoxicity of PARP inhibitors was dramatically enhanced by mTOR inhibitors in BRCA-proficient triple-negative breast cancers *in vitro* and *in vivo*, at the same time revealing a novel mechanism for mTOR signaling to regulate the homologous recombination process (39). The role of PARP-1 in autophagy and tumorigenesis is worthy to be studied further and PARP1 inhibitors might have potential efficacy in LAM treatment.

## Receptor Tyrosine Kinases and Non-receptor Tyrosine Kinases

RTKs are a critical group of cell surface receptors located upstream of PI3K/Akt/mTORC1 pathway to receive and transfer growth factors including PDGF, VEGF-C, and VEGF-D, activating several downstream signal pathways to regulate tumor progression. Overexpression of various types of RTKs such as epidermal growth factor receptors (EGFRs), vascular endothelial growth factor receptors (VEGFRs), platelet-derived growth factor receptors (PDGFRs), and insulin-like growth factor receptors (IGFRs) is found in different types of cancer including LAM (40–42), indicating the important role of RTKs in LAM. The inhibition of RTKs is proposed to block the mTOR pathway. Studies proved its feasibility when they found that anti-EGFR antibody caused progressive cell death in TSC2^−/−^ and TSC2^−^/meth cells (TSC2^−^/meth cells refer to smooth muscle-like cells from AML cells for these cells are methylated in the TSC2 promoter, and their proliferative, morphological, and biochemical characteristics are very similar to TSC2^−/−^ smooth muscle cells) (43). Subsequently, the *in vivo* study showed that numbers of mice with lung nodules and the average area of the nodules was significantly reduced by Ab treatment and in combination with rapamycin (44). Moreover, angiogenesis and lymphangiogenesis reduction and even lung degeneration reservations were observed in Ab treatment (44). Another typical RTK, VEGFR, expressed on cancer cells, endothelial cells, and other stromal cells is found to be related to tumor lymphangiogenesis, angiogenesis, and growth (45). A study showed that axitinib, a small molecule tyrosine kinase inhibitor that targets VEGFR, attenuated VEGF-D upregulation in serum and lung lining fluid, as well as inhibited Tsc2-null lung lesion growth and abnormal lymphangiogenesis in a mouse model of LAM (42). It suggested that targeting this receptor may be beneficial for the treatment of LAM, especially considering that VEGF-D is a critical serum biomarker for LAM diagnosis and for evaluating the severity of LAM and efficacy of treatment in the presence (46, 47). A well-studied RTK inhibitor is nintedanib that dose-dependently inhibits PDGFR phosphorylation and has been used to treat IPF. Based on these data, a clinical trial assessing the efficacy, safety, and tolerability of nintedanib in the treatment of LAM is being carried on (Table 1).

A classic representative of non-RTKs is the Src family, which is also involved in the regulation of extracellular regulated protein kinases (ERK) and PI3K activation (Figure 1), modulating the equilibrium between survival and apoptosis, and might be affecting cystic fibrosis (48). The phosphorylation of Src is regulated by C-terminal Src kinase (Csk), a cytoplasmic protein–tyrosine kinase, which possesses a greater affinity for Src when recruited to the membrane by a transmembrane Csk binding protein (Cbp) than the free state (49) (Figure 1). A study has found upregulated phosphorylation of Src on Tyr416 in lung tissues of LAM patients compared with normal tissues, and enhanced activation of Src-kinase signaling pathway resulted from autophagy inhibition was observed *in vitro* studies in which Src inhibition of saracatinib reduced the epithelial–mesenchymal transition (EMT) which is promoted by overexpression of Src in TSC-deficient cells (50). EMT is believed to be correlated with infiltrative growth pattern, metastatic potential, and altered cell differentiation of LAM cells. Moreover, saracatinib was proved to attenuate migration and invasion activity of TSC2^−/−^ cells in an *in vitro* study and reduce lung colonization in an *in vivo* study, suggesting that Src inhibition could reduce the metastatic potential for TSC2^−/−^ cells (50). Then, a clinical trial studying the safety and efficacy of saracatinib in patients with lymphangioleiomyomatosis is currently being conducted (Table 1).

Spleen tyrosine kinase (Syk) is another non-RTK that has a key role in the immune system but arousing much concern in tumorigenesis study recently (51). It had an aberrant expression in various malignant tumors and contributing to the initiation and metastatic progression (52, 53). Phosphorylation of Syk is involved in the activation of the PI3K/Akt signaling pathway, mainly transducing immune response-associated signaling (54) (Figure 1). It is also a regulator of mTOR because its depletion resulted in a significant reduction of S6K1 phosphorylation in follicular lymphoma cells (55). Hence, the hypothesis that Syk may be involved in LAM pathogenesis is worthy to be taken into consideration. As expected, deregulated expression and activation of Syk was detected in TSC2-deficient cells and LAM lung lesions (56). Furthermore, Syk inhibitor fostamatinib reduced the proliferation of TSC2-deficient cells *in vitro* and suppressed TSC2-null xenograft tumor development *in vivo* (56). This study identified a Syk-dependent signaling inducing VEGF-D expression in peripheral blood mononuclear cells by monocyte chemoattractant protein (MCP)-1, which was elevated via signal transducer and activator of transcription (Stat3) signaling downstream of the mTORC1 signaling (56), demonstrating the strong therapeutic potential of Syk in LAM treatment. Fostamatinib, as a Syk inhibitor, has been approved by the FDA to treat immune thrombocytopenia, but clinical trials studying its efficacy and safety in LAM treatment still wait to be accomplished.

## Hormones and Compensatory ERK Pathway

As is illustrated that LAM happens exclusively in women and is exacerbated during pregnancy, roles of female hormones in the development and progression of LAM call for more studies, and the evidence does suggest the relationship between female hormones and LAM pathogenesis. An immunohistochemistry study found progesterone receptor (PR) and estrogen receptor (ER) immunoreactivity in the smooth muscle component of renal angiomyolipomas (100 and 83%) from 12 LAM patients (57), and prevalence of PR expression over ER was confirmed in a large series of pulmonary LAM cases (58); in *in vitro* studies, ER-α and ER-β expression was observed in cultured angiomyolipoma cells derived from LAM patients (59).

Estrogen has been reported to promote changes in gene expression and can induce the activation of signaling proteins such as Src, Akt, and ERK (60) (Figure 1). It was proposed that the estrogen-regulated ERK pathway and the constitutive mTORC1 pathway together promote migration and invasion in LAM patient–derived cells by converging on the late response genes product (Fra1)–zinc finger E-box-binding homeobox (ZEB)-1/2 transcriptional network (61) (Figure 1). Studies already observed the stimulation effect of estradiol and tamoxifen on the growth of cultured angiomyolipoma cells (59). It was found that when the tuberin function is lost, the proliferation of Eker rat uterine leiomyoma derived smooth muscle (ELT3) cells promoted by estrogen is associated with the activation of PDGFR and ERK pathway (62). Subsequent studies found that estrogen promoted lung colonization of Tsc2-null ELT3 cells in pulmonary metastasis, which could be blocked by mitogen-activated protein kinase kinase (MEK) inhibitors CI-1040 in *in vivo* studies (63). In addition, a recent study found that estrogen increases membrane translocation of glucose transporters and enhances glucose uptake in a PI3K/Akt-dependent way in mTORC1 hyperactive cells (64). Estrogen promotes glucose metabolism including upregulating the expression of glucose-6-phosphate dehydrogenase (G6PD) and increasing nicotinamide adenine dinucleotide phosphate (NADPH) and ROS production, and thereby promotes cell survival under oxidative stress (65). A randomized controlled trial of letrozole (a non-steroidal, competitive aromatase inhibitor lowering serum estrone and estradiol) for postmenopausal women with LAM showed that the rate of change for serum VEGF-D between placebo group and letrozole group was −0.024±0.009 pg/ml/month (*P* = 0.015), but the rate of change in FEV_1_ for all subjects was −3 ± 3 ml/month (*P* = 0.4) (66). Considering the small size (15) of the trial, more evidence is required to prove the effects of hormonal treatment in LAM.

The study on estrogen still revealed the possibility of targeting the ERK pathway as a therapy in LAM treatment. ERK belongs to mitogen-activated protein kinases (MAPKs) family and plays a key role in tumorigenesis including cancer cell proliferation, migration, and invasion through cascade Ras/Raf/MEK/ERK, as a critical compensatory signaling pathway of PI3K/Akt/mTOR (67) (Figure 1). In the ERK signaling pathway, Ras, a membrane-associated GTP-binding protein, binds and activates several effector proteins including PI3K and rapidly accelerated fibrosarcoma (Ras) when activated by RTKs (68). ERK1/2 then is activated by MEK1/2 as a result of the regulation of RAF (67) (Figure 1). The Ras/Raf/MEK/ERK and PI3K/Akt/mTOR pathways can cross-regulate each other's activity. For instance, ERK inhibits the recruitment of PI3K when induced by EGF and promotes mTORC1 directly or through repressing TSC1/2 complex; IGF1 stimulation could result in negative regulation of Ras/Raf/MEK/ERK pathway by inducing Akt inhibiting Raf (69) (Figure 1). In recent years, it has been reported that inhibition of mTORC1 induced activation of Ras/Raf/MEK/ERK signaling in renal cell carcinoma, prostate cancer, and leiomyosarcomas (70–72). In addition, MEK inhibitor as a melanoma treatment is now attractive for the treatment of non–small cell lung carcinoma (NSCLC) for its anti-cancer activity in patients with NSCLC (73). Considering the compensatory nature of the ERK pathway, whether MEK inhibitor in combination with rapamycin would benefit LAM patients requires to be studied carefully.

The unusual high PR/ER ratio detected by immunohistochemistry in renal angiomyolipomas reveals the possible function of progesterone in LAM (58). Progesterone is well–known to be antiproliferative for uterine leiomyomas, leading to the assumption of its anti-tumor function. Previous studies found that progesterone therapy stabilized chylous pleural effusion and ascites in some LAM patients (74). Nevertheless, retrospective studies showed less decline of lung function level in patients receiving progesterone therapy than in those who were not treated with progesterone but with no significance (75, 76), which seems to suggest that patients with LAM may not benefit from this therapy. Conversely, in *in vitro* studies, progesterone was found to activate ERK1/2 and Akt pathways, as well as enhance the proliferation, migration, and invasiveness of TSC2-deficient cells (77). Therefore, the specific mechanism of how estrogen and progesterone affect LAM development and their therapeutic potential still requires further exploration.

## EMT Process in LAM Metastasis

The dissemination of tumor cells from the primary site to several new sites in the body is typical in a malignant tumor, and so is it in LAM progression. Genetic confirmation of recipient origins of recurrent LAM lesions within the donor allografts of LAM patients, who had received lung transplantation, validates the metastasis theory for LAM, which is also indicated by multiple systemic manifestations and anatomy evidence (1). Therefore, to illuminate and prevent the metastasis process is particularly important and of increasing interest as a therapeutic avenue in the treatment of LAM.

The EMT has been proposed as a critical mechanism during cancer progression and metastasis because it is believed that the transition from epithelial to mesenchymal states allows a cell to break away from its originating tissue, traveling throughout the body (78). Numerous signaling pathways are involved in EMT induction, including PI3K/Akt pathway in which nuclear factor κB (NFκB) activated by Akt induces SNAIL1 stabilization that plays an integral role throughout EMT, and ZEB proteins which were mentioned earlier to engage the integration of mTORC1 and ERK pathway (78) (Figure 1). Among this complex pathway network inducing EMT, a protein family consisting of ezrin, radixin, and moesin (ERM) has come into focus of attention in investigations regarding metastatic cancer.

Dynamic rearrangement of the cytoskeleton is crucial to cell mobility and migration in metastasis, and ezrin, radixin, and moesin are important membrane-cytoskeletal crosslinkers which are suggested to play vital roles in cancer progression, especially ezrin and moesin (79). Studies have found that upregulated expression of moesin was associated with higher histological grade, advanced stage, and poor prognosis in gastric adenocarcinoma, breast carcinoma, and oral squamous cell carcinomas (79–81). Early in 2002, abundant expression of ezrin and moesin was observed in TSC-associated cortical tubers (82). Further studies showed that the role of ERM in tumor metastasis might result from a mTORC2-dependent pathway in which mTORC2 is found to modulate the actin cytoskeleton and promote migration and invasiveness through regulating RhoA activity, as is demonstrated previously (Figure 1). More specifically, GTPase-loaded RhoA activates Rho-associated protein kinases (ROCK1 and ROCK2) to promote the formation of stress fibers and the inhibition of cofilin and actin, supporting an amoeboid migration instead of a mesenchymal one, both morphologies being adopted during single-cell migration (78) (Figure 1). In the intravasation stage of metastasis, RhoA/ROCK provides cellular contractility to produce major cell deformability, enabling the cell to radically adjust its cytoskeleton to squeeze between intercellular spaces (78). As we expected, moesin is found to be a requirement to activate RhoA signaling in response to initial attachment and spreading (83), highlighting the urge to study the specific mechanism of how ERM, EMT, and mTORC2 integrally affect LAM metastasis.

**Figure 3:**
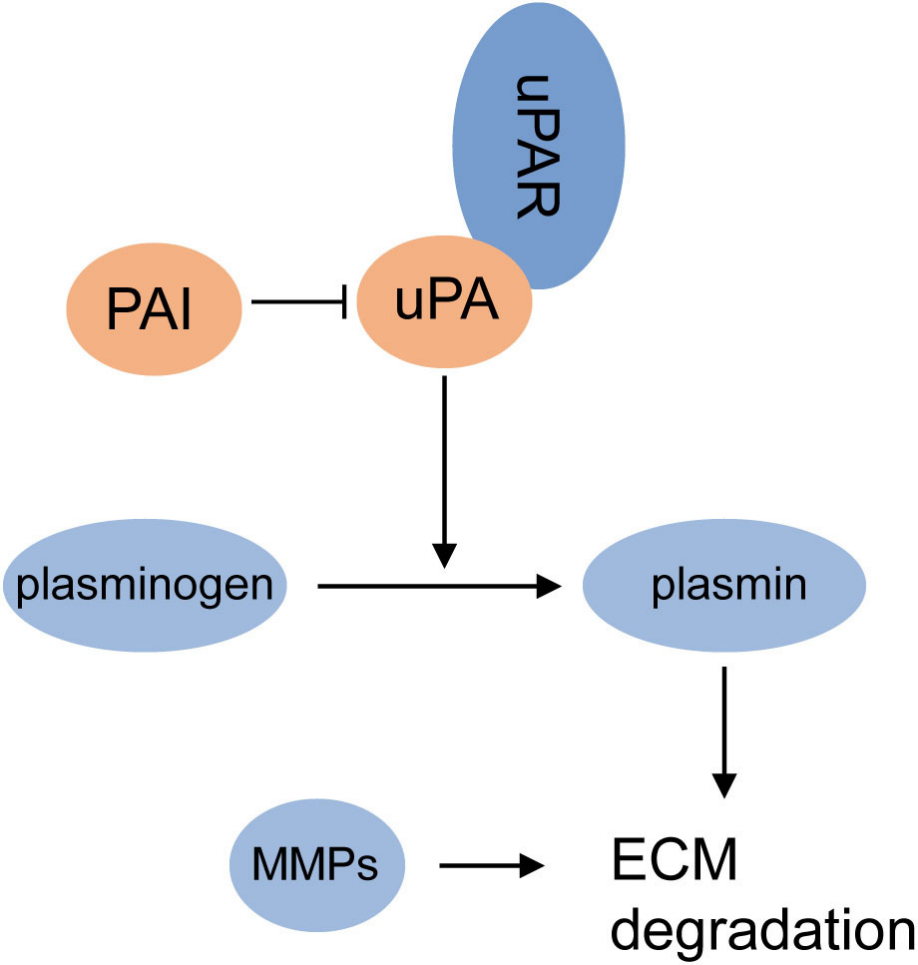
uPA system and MMPs promote ECM degradation. uPA is recruited by uPAR and is negatively regulated by PAI1 or PAI2; overproduction of uPA increases the transformation from plasminogen to plasmin; plasmin promotes the degradation of ECM surrounding the tumor with MMPs, and promotes the initiation of invasion and metastasis.

Urokinase-type plasminogen activator (uPA) is a serine protease that also plays a role in tumor invasive process, at the same time upregulated in LAM lesions and angiomyolipomas. The uPA system, including uPA, plasminogen activator inhibitor (PAI)-1, PAI-2, and uPA-associated receptor (uPAR), regulates the production and function of uPA to modulate the conversion from plasminogen to plasmin, which promotes the proteolytic cleavage of extracellular matrix (ECM) surrounding the tumor with matrix metalloproteinases (MMPs), facilitating the detachment of tumor cells from an original site and the initiation of invasive and metastasis (84) (Figure 3). The abnormal expression of the uPA system has been detected in breast cancer, prostate cancer, ovarian cancer, and lung cancer (84), and was recently reported in TSC-deficient tumors. The study found that overexpression of uPA was a direct consequence of TSC mutation in LAM cells and mTOR inhibitors even increased expression of uPA in cells with compromised TSC function (85). However, inhibition of uPA expression in TSC2-null tumor cells could not only reduce their invasive and mitogenic potentials but also increase their susceptibility to the pro-apoptotic agent simvastatin (85). Together, these studies indicate that uPA might serve as a potential target to prevent the dissemination of tumor cells and the progression of disease in LAM treatment.

## Newly Found Potential Targets

### Function of mTORC2

As mentioned previously, mTORC2 is rapamycin insensitive, and the function of mTORC2 in LAM pathogenesis remains unclear, however, which does not mean that mTORC2 contributes little in tumorigenesis. Further study into the mechanism of the mTORC2 pathway might find novel targets improving the treatment of LAM. Rictor, a GTPase, is another component of mTORC2 besides mTOR (Figure 1). siRNA-mediated knockdown of rictor presents thick actin fibers throughout much of the cytoplasm and less prominent actin in HeLa cells, and the pattern of the actin staining is similar to that in cells with reduced mTOR expression, indicating the regulation effect of rictor and mTOR in organization of the actin cytoskeleton (86). Similarly, siRNA rictor microinjected in TSC^−/−^ MEFs is found to attenuate the increased stress fiber formation as well as the enhanced migration and invasiveness of cells, whereas rapamycin has little effect on migration of LAM cells (87). These studies indicated the possibility of targeting mTORC2 as a therapy in combination with rapamycin.

The statin family of pharmacological inhibitors have been proposed as a treatment for LAM and examined in several clinical trials. Atorvastatin and simvastatin separately target farnesylation of Rheb and RhoA activity (88), and showed distinct effects in preclinical *in vivo* studies in which atorvastatin did not reduce tumor growth or improve survival in a Tsc2+/– mouse model with kidney and liver tumors while simvastatin inhibited tumor growth in a model of TSC2-null subcutaneous tumors (14, 89). The effects to preventing TSC2-null tumor growth and inhibiting destruction of the lung tissue were enhanced when simvastatin was in combination with rapamycin (90). The better effect of simvastatin may come from its target, RhoA, through which the mTORC2 could regulate the actin cytoskeleton (Figure 1), revealing the unneglected role of mTORC2 in tumor migration and invasiveness. A clinical trial studying the safety of simvastatin in patients with either LAM or TSC who are already being treated with rapamycin was just completed (Table 1), and we are looking forward to its outcome.

In addition to affecting cell migration, mTORC2 may also play a part in cell growth in some manner. An immunohistochemical study evaluating the expression of IGFs, IGF-1R, and IGFBPs in the lungs of patients with LAM found that only a proportion of patients have IGF-1 expression in LAM cells while reaction for IGF-2 was detected in almost every patient enrolled (40). The abnormal expression of IGFs in LAM patients may be related to mTORC2. A study reported that increased level of insulin, IGF-1, and IGFBP-3, which is a major IGF1 binding protein that stabilizes IGF-1 in the serum, was observed in adipose-specific Rictor knockout mice (rictor ad^−/−^) that have gained weight due to an increase in the size of non-adipose tissue (91). These results suggested that mTORC2 might affect cell growth through regulating IGF-1, but the specific mechanism is yet to be demonstrated.

### DNA Damage Checkpoint

**Figure 4:**
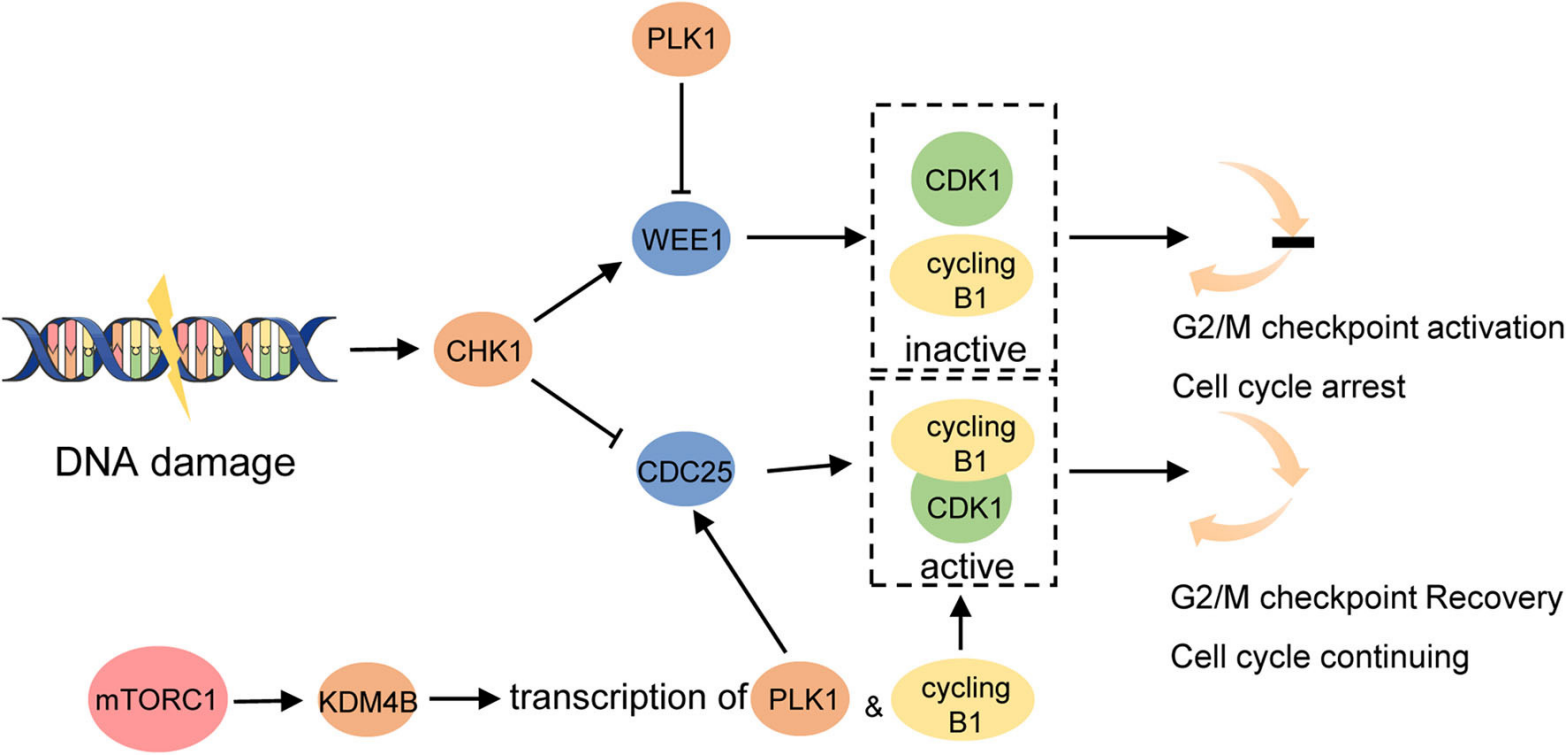
mTORC1 promotes G2/M checkpoint recovery triggered by DNA damage. DNA damage activates CHK1. It then activates WEE1 which maintains cyclin B1 complexed with CDK1 inactivated and prevents cell cycle from entering mitosis. CHK1 inhibits CDC25 at the same time which activates CDK1/cyclin B1 complex and promotes G2/M checkpoint recovery. Hyperactivated mTORC1 increases the function of KDM4B which increases the transcription of cyclin B1 and PLK1. PLK1 activates CDC25 and inhibits WEE1 to promote the cell cycle to continue.

The DNA damage checkpoints trigger cell-cycle arrest, providing sufficient time for the repairment of damaged chromosomes to help prevent the segregation of damaged or mutated chromosomes and promote genomic stability (92). G2/M is the last checkpoint before mitosis which is preferred in cancer cells to repair DNA damage and is regulated by the status of cyclin-dependent kinase 1(CDK1)/cyclin B1 complex (93). DNA damage is detected by related protein kinases and activates cell-cycle checkpoint kinase 1 (CHK1) which then phosphorylates cell division cycle 25 homolog (CDC25) and WEE1, repressing CDC25 and activating WEE1 at the same time. Because WEE1 negatively regulates CDK1/cyclin B1 while CDC25 does conversely, these physiological changes together contribute to the inhibition of CDK1/cyclin B1 complex and trigger the mitosis pause (93) (Figure 4). To study the signaling network required for cell-cycle restart, a systems biology approach has recently identified the essential role of mTOR signaling in regulating the recovery from G2/M checkpoint. The study found that transcription of cyclin B1 and polo-like kinase 1 (PLK1) was significantly impaired when DNA gets damaged in mTOR-depleted cells, which provided a suppressive chromatin environment due to decreased KDM4B function (94). As we know, PLK1 has the opposite effect with CHK1 which upregulates CDC25 but downregulates WEE1, helping promote the entry of mitosis (95) (Figure 4). This result indicates that in mTOR-activated cells, like LAM cells, cell proliferation seems to be enhanced because of a shorter G2/M checkpoint pause compared with normal cells, leaving insufficient time for DNA repair. In the subsequent study, TSC2-null cells did exhibit accelerated G2/M checkpoint recovery. Furthermore, these cells are sensitive to WEE1 inhibitors MK1775 both in *in vitro* and *in vivo* studies, particularly, more sensitive to rapamycin combined with the WEE1 inhibitor compared with rapamycin alone (94). WEE1 is a key nuclear kinase that is a crucial component to trigger G2/M checkpoint arrest and was reported to be involved in cancer drug resistance. For instance, WEE1 expression was increased after EGFR-TKI resistance in NSCLC and the combination of chemotherapy with WEE1 inhibitor might improve the clinical outcome of NSCLC patients with acquired EGFR-TKI resistance (96). Moreover, a recent study in patients with locally advanced pancreatic cancer demonstrated prolonged overall survival with the addition of the Wee1 kinase inhibitor adavosertib to gemcitabine plus radiotherapy (97). These studies provide a new opportunity for targeting tumor cells with mTORC1 hyperactivation like LAM using WEE1 inhibitors to induce mitotic catastrophe and cell death.

### NR2F2 Located by GWAS in S-LAM Patients

Unlike TSC-LAM, the origin of S-LAM is not fully elucidated. The inactive mutations are seen in most LAM lesions but not in the germline of S-LAM patients. Researchers proposed a hypothesis that DNA sequence variants outside of TSC1/TSC2 might be associated with disease risk, and they applied genome-wide association studies (GWASs) to validate the assumption through analysis of over 5.4 million single-nucleotide polymorphisms in 426 S-LAM DNA samples and genotype data of 852 healthy female volunteers obtained from COPDGene project (98). This study finally identified a signal on chromosome 15q26.2 that reached genome-wide significance adjacent to the gene encoding nuclear receptor subfamily 2, group F, member 2 (NR2F2), which belongs to the steroid receptor superfamily (99). Its expression was higher in LAM and angiomyolipoma by RNA sequencing analysis in comparison with large cancer and normal tissue datasets (98). Studies have suggested that NR2F2, also known as chicken ovalbumin upstream promoter transcriptional factor II (COUP-TFII), serves as a major regulator to control angiogenesis within the tumor microenvironment and tumor lymphangiogenesis through cooperation with VEGF/VEGFR signaling, although the specific details need to be further elucidated (100, 101). VEGF-D serves as a diagnostic biomarker for LAM and possible driver for lymphangiogenesis. In TSC-null cells, inappropriate activation of mTOR results in accumulation of hypoxia-inducible factor-1α (HIF-1α) and increased expression of HIF-responsive genes including VEGF, which could be decreased by rapamycin treatment (102) (Figure 1). In addition, a study found that hypoxia significantly downregulated COUP-TFII in early endothelial progenitor cells (103). Further investigation may help reveal the interaction between COUP-TFII, HIF-1α, and VEGF. Previous studies have already shown that the siRNA knockdown of ERα in MCF-7 breast cancer cells decreased COUP-TFII expression while treatment with estradiol increased the expression of COUP-TFII, revealing a positive correlation between COUP-TFII and ERα status (104). A recent study also reported another mechanism of how COUF-TFII might influence LAM pathogenesis, in which lactate production induced by upregulated COUP-TFII in KRAS-activated cells increases mTORC1 activity by disrupting the interaction of TSC2 and Rheb (105) (Figure 1). Thus, NR2F2 could be a promising candidate to explain the gene origin of S-LAM and pathogenesis of LAM.

## Conclusion

Despite the rare nature of this disease, the study into tumorigenesis, migration, and invasiveness of LAM has made tremendous advances in the past 20 years. The activation of mTOR signaling, regulated by inactivation mutation of TSC2, is the key point of LAM pathogenesis and the crucial target of rapamycin therapy, which is a hallmark event in LAM treatment. Nevertheless, rapamycin therapy is not curative and studies about other potential targets have been carried out. Among these hypotheses, autophagy induced by rapamycin may explain its limited efficacy and reveal the probable benefit of autophagy inhibitors. Common methods used in finding and validating new targets usually start from upstream or downstream pathways affecting PI3K/Akt/mTORC1 signaling, like tyrosine kinases, or compensatory pathways independent of mTORC1 signaling, like mTORC2 pathway, modulating actin cytoskeleton, and ERK pathway, related with estrogen signaling. However, they may also coordinate with mTORC1 signaling. Considering the characteristic damage caused by LAM to the lung tissue and pulmonary function, studying the cyst formation mechanism is of significance to prevent the parenchymal destruction of lung and improve the general condition of LAM patients. Another vital process in LAM progression is metastasis in which the EMK process might contribute a lot to the migration and invasiveness of LAM cells. Besides, new methods such as GWAS, which located a new gene NR2F2 involved in LAM, and systems biology approach, which found the correlation of mTOR with DNA damage checkpoint, used in tumor research could help open minds in studying new targets, which, however, need to be further validated. What is more, some of the mechanisms demonstrated in this review, although illustrated from a different angle, present a potential relation. For example, the little-understood mTORC2 pathway is also found to affect the migration of LAM cells by regulating RhoA signaling, which plays a key role in the EMT process. Therefore, studying the integration of related pathways may help uncover the pathogenesis of this complex disease and find an effective therapy.

## Author Contributions

LY and MJ contributed to the conception and design of the study. XS and HC organized the database and wrote the article. All authors contributed to article revision, and read, and approved the submitted version.

## Conflict of Interest

The authors declare that the research was conducted in the absence of any commercial or financial relationships that could be construed as a potential conflict of interest.
